# The effects of the exposure to neurotoxic elements on Italian schoolchildren behavior

**DOI:** 10.1038/s41598-021-88969-z

**Published:** 2021-05-10

**Authors:** Stefano Renzetti, Giuseppa Cagna, Stefano Calza, Michele Conversano, Chiara Fedrighi, Giovanni Forte, Augusto Giorgino, Stefano Guazzetti, Costanza Majorani, Manuela Oppini, Marco Peli, Francesco Petrucci, Anna Pino, Donatella Placidi, Oreste Senofonte, Silvia Zoni, Alessandro Alimonti, Roberto G. Lucchini

**Affiliations:** 1 Department of Occupational Health, University of Brescia, Piazzale Spedali Civili, 1, 25123 Brescia, BS Italy; 2 Department of Molecular and Translational Medicine, University of Brescia, Brescia, Italy; 3 Department of Public Health, ASL, Taranto, Italy; 4 Department of Environment and Health, Italian National Institute of Health, Rome, Italy; 5 Cosmetics and Consumer Protection, Italian National Institute of Health, National Centre for Chemicals, Rome, Italy; 6 Department of Public Health, Azienda USL IRCCS di Reggio Emilia, Reggio Emilia, Italy; 7 Department of Civil, Environmental, Architectural Engineering and Mathematics, University of Brescia, Brescia, Italy; 8 Florida International University, Miami, FL USA

**Keywords:** Human behaviour, Risk factors

## Abstract

Neurodevelopmental disorders are constantly increasing on a global scale. Some elements like heavy metals are known to be neurotoxic. In this cross-sectional study we assessed the neurobehavioral effect of the exposure to trace elements including lead, mercury, cadmium, manganese, arsenic and selenium and their interactions among 299 schoolchildren residing in the heavily polluted Taranto area in Italy. Whole blood, urine and hair were collected for metal analyses, while the Child Behavior Checklist and the Social Responsiveness Scale, administered to the main teacher and the mothers were considered to identify behavioral problems in children. Blood lead mainly influenced social problems, aggressive behavior, externalizing and total problems. Urinary arsenic showed an impact on anxiety and depression, somatic problems, attention problems and rule breaking behavior. A significant interaction between lead and arsenic was observed, with a synergistic effect of the two metals increasing the risk of attention problems, aggressive behavior, externalizing problems and total problems. Overall, we were able to test that higher blood lead, urinary arsenic concentrations and their interaction increase the risk of neurobehavioral problems. This is in line with the U.S. Environmental Protection Agency’s priority list of hazardous substances where arsenic and lead are ranked as first and second respectively.

## Introduction

Neurodevelopmental disorders including autism, Attention Deficit Hyperactivity Disorder (ADHD), learning disabilities and behavior, affect 10–15% of the births^1^ and are constantly increasing on a global scale^2^. Similar results were observed among US children^3^. In two Regions of Central Italy, about 1.3% of the Italian elementary and middle-school children were reported to suffer from severe ADHD^4^. According to the statistics on the incidence of autism from the U.S. Centers for Disease Control and Prevention (CDC), one child every 6–8 suffers from autistic spectrum disorders in the USA^5^. A comparison in temporal trends showed that the prevalence of diagnosed autism has risen dramatically in the U.S over the last several decades, mostly since the 1980s, and continues to trend upward. Average prevalence in Asia, Europe and North America varies between 1 and 2%^6^. These data have shown a tenfold increase in the last 40 years about 3% of developmental disabilities may be a consequence of environmental exposure to neurotoxicants and another 25% of the interaction between environmental hazards and individual genetic predisposition^7,8^. Environmental toxicants show increasing trends that correlate positively to the rise in autism^9^. Neurotoxic elements including lead (Pb), mercury (Hg), cadmium (Cd), manganese (Mn) and arsenic (As) are known being able to cross the placenta through sequence variations which can affect protein metabolizing and transporting metals^10^ and to cross the blood–brain barrier and interfere with neurons^11,12^ while selenium (Se) is a potentially protective agent for metabolic defense against oxidative stress, although its margin of safety is rather narrow^13,14^. Several studies on children environmentally exposed to these elements have shown impacts on attention and behavior and school performance^15–26^. Few works exploring behavioral effects of the exposure to metal mixture have found an increased harmful effect^27–30^. However, due to the complexity of the problem more evidences are needed about the synergistic effect of the exposure to metals on neurobehavior. The exact neurotoxicological mechanism through which a mixture of metals including Pb, Hg, Cd, Mn and As can lead to behavioral problems is still unclear and there is the need of more investigation and evidences on how metal mixtures and the interactions among the chemicals can influence behavior^31^.

In this study we assessed the neurobehavioral effect of the exposure to some elements including Pb, Hg, Cd, Mn, As and Se among 6–11 years old children residing in the heavily polluted Taranto area in Italy considering the interactions among the elements that showed a significant association with the behavioral outcomes.

## Results

Table 1 shows the overall socio-demographic characteristics of participants, grouped also by the area of residence. Two of the neighborhoods closer to the industrial site (Tamburi and Paolo VI) were characterized by a lower percentage of high-level socio-economic status (SES) (1.3% and 0% respectively, Chi-squared p-value < 0.001), a higher frequency of passive smoking exposure (26.9% and 11.1% respectively, Chi-squared p-value < 0.001) and lower Intelligence Quotient (IQ) (median IQ equal to 94 and 102 respectively, compared to 110 in the neighborhoods of Statte and Talsano, Kruskall–Wallis p-value < 0.001) Children residing in Tamburi also showed lower cognitive stimulation at home (median (1Q-3Q) HOME total score equal to 5.0 (4.0, 6.0), Kruskall-Wallis p-value < 0.001), were slightly older and had a higher percentage of females than children living farther from the source.

**Table 1:** Overall and stratified by area of residence socio-demographic characteristics.

	Total (N = 299)	Tamburi (N = 78)	PaoloVI (N = 27)	Taranto (N = 59)	Statte (N = 30)	Talsano (N = 105)	p value
**N (%)**							
Sex							0.154
F	161 (53.8%)	51 (65.4%)	11 (40.7%)	30 (50.8%)	15 (50.0%)	54 (51.4%)	
M	138 (46.2%)	27 (34.6%)	16 (59.3%)	29 (49.2%)	15 (50.0%)	51 (48.6%)	
**SES**							< 0.001
Low	124 (41.5%)	55 (70.5%)	13 (48.1%)	12 (20.3%)	14 (46.7%)	30 (28.6%)	
Medium	103 (34.4%)	22 (28.2%)	14 (51.9%)	16 (27.1%)	8 (26.7%)	43 (41.0%)	
High	72 (24.1%)	1 (1.3%)	0 (0.0%)	31 (52.5%)	8 (26.7%)	32 (30.5%)	
**Passive smoking**							< 0.001
No	262 (88.2%)	57 (73.1%)	24 (88.9%)	57 (96.6%)	30 (100.0%)	94 (91.3%)	
Yes	35 (11.8%)	21 (26.9%)	3 (11.1%)	2 (3.4%)	0 (0.0%)	9 (8.7%)	
**Median (1Q-3Q)**							
Age (years)	8.8 (7.3, 9.9)	9.5 (7.3, 10.3)	8.8 (7.6, 9.3)	8.6 (7.0, 9.4)	8.1 (7.0, 8.9)	8.8 (7.3, 10.2)	0.005
Distance (km)	5.8 (1.4, 10.0)	0.9 (0.7, 1.1)	5.1 (5.1, 5.5)	5.1 (4.1, 5.7)	7.8 (7.6, 8.2)	11.1 (10.0, 11.6)	< 0.001
IQ	106.0 (94.0, 115.0)	94.0 (85.0, 107.0)	102.0 (93.0, 114.0)	106.0 (96.5, 117.0)	110.0 (99.5, 116.5)	110.0 (101.0, 118.0)	< 0.001
HOME	6.0 (4.0, 7.0)	5.0 (4.0, 6.0)	6.0 (3.5, 6.5)	6.0 (5.5, 7.0)	5.0 (4.0, 6.0)	6.0 (5.0, 7.0)	< 0.001

Overall and stratified by area of residence socio-demographic characteristics. Chi-squared test and Kruskal–Wallis rank-sum test were performed to test differences among the areas of residence for categorical and continuous variables respectively.

Metal concentrations showed a low inter-correlation (Table S1 of the Supplementary Material) with the highest value equal to 0.276 corresponding to the correlation between urinary As (UAs) and hair Hg (HHg).

After checking the assumption of a linear relationship through cubic splines (data not shown), we observed a negative trend of metal concentrations at increasing distance from the source of exposure (Table 2). Only HHg and blood Se (BSe) showed a positive association.

**Table 2:** Biomarkers descriptive statistics.

	Median (Q1, Q3)	β coeff (95% CI)
UAs (ng/mL)	8.3 (5.1, 15.1)	− 0.04 (− 0.06, − 0.01)
UCd (ng/mL)	0.4 (0.3, 0.7)	− 0.02 (− 0.05, − 0.001)
HHg (ng/g)	476.6 (271.1, 744.7)	0.04 (0.02, 0.07)
HMn (ng/g)	135.3 (87.9, 202.4)	− 0.02 (− 0.05, − 0.003)
BPb (ng/mL)	8.4 (6.4, 11.0)	− 0.001 (− 0.01, 0.01)
BSe (ng/mL)	142.7 (126.8, 161.0)	0.01 (0.001, 0.01)

Biomarkers descriptive statistics and association with distance from the emission source (β coefficients and 95% CI).

A poor agreement was found between parents and teachers evaluation (ICC ranging from 0.06 for anxiety problems to 0.36 for conduct problems) while the skewness and kurtosis highlighted a non-normal distribution of the lower level empirically-based syndrome and the DSM oriented scales (skewness and kurtosis above 0.5 and 3 respectively for all these outcomes). Tables 3 and 4 show median and 1st–3rd quartiles interval of the neurobehavioral scores including the CBCL empirically based and DSM oriented scales reported by parents and teachers respectively. Higher scores indicate higher levels of problems in an area meaning better performance in the more distant areas while lower scores indicate fewer problems. The p-value of the Kruskal–Wallis is also reported.

**Table 3:** Total and divided by area parent CBCL scales and SRS total score.

	Tamburi (N = 78)	PaoloVI (N = 27)	Taranto (N = 59)	Statte (N = 30)	Talsano (N = 105)	Total (N = 299)	p value
**Empirically based scales**							
Anxious/depressed	57.0 (51.2, 63.0)	57.0 (51.0, 64.5)	57.0 (52.0, 64.0)	62.0 (53.0, 65.0)	57.0 (51.0, 66.0)	57.0 (51.0, 65.0)	0.968
Withdrawn/depressed	58.0 (52.0, 62.0)	54.0 (52.0, 64.0)	56.0 (50.0, 62.0)	56.0 (52.0, 61.5)	56.0 (50.0, 60.0)	56.0 (50.0, 62.0)	0.968
Somatic complaints	55.0 (50.0, 60.0)	53.0 (53.0, 61.0)	53.0 (50.0, 61.0)	57.0 (50.8, 61.0)	53.0 (50.0, 61.0)	53.0 (50.0, 61.0)	0.968
Social problems	**54.0 (53.0, 59.0)**	**52.0 (51.0, 57.5)**	**52.0 (51.0, 56.5)**	**57.0 (53.2, 59.8)**	**53.0 (51.0, 57.0)**	**54.0 (51.0, 58.0)**	**0.037**
Thought problems	51.0 (51.0, 54.0)	54.0 (51.0, 54.0)	54.0 (50.0, 56.0)	52.5 (51.0, 58.0)	51.0 (50.0, 54.0)	51.0 (50.0, 54.0)	0.968
Attention problems	53.0 (51.2, 59.0)	52.0 (51.0, 55.0)	53.0 (51.0, 59.0)	54.0 (51.0, 60.5)	52.0 (51.0, 55.0)	53.0 (51.0, 57.0)	0.968
Rule-breaking behavior	52.0 (51.0, 58.5)	52.0 (50.5, 57.0)	52.0 (51.0, 57.0)	52.5 (51.0, 58.5)	52.0 (50.0, 55.0)	52.0 (50.0, 57.0)	0.903
Aggressive Behavior	54.5 (50.0, 59.0)	52.0 (50.0, 55.5)	52.0 (50.0, 56.0)	55.5 (51.2, 59.0)	52.0 (50.0, 57.0)	52.0 (50.0, 57.0)	0.827
Internalizing broad band score	58.0 (50.0, 63.0)	57.0 (50.0, 65.0)	58.0 (51.0, 63.0)	61.0 (56.2, 64.0)	57.0 (50.0, 63.0)	58.0 (50.0, 63.0)	0.968
Externalizing broad band score	54.0 (47.2, 58.0)	50.0 (44.0, 55.0)	51.0 (47.0, 56.0)	54.0 (50.2, 58.0)	50.0 (46.0, 56.0)	51.0 (47.0, 57.0)	0.501
Total problems score	55.0 (50.0, 59.8)	53.0 (47.5, 59.0)	53.0 (48.5, 59.0)	58.5 (52.5, 60.8)	52.0 (46.0, 59.0)	54.0 (48.0, 59.0)	0.827
**DSM-oriented scales**							0.968
Affective problems	58.0 (52.0, 63.0)	52.0 (52.0, 58.0)	56.0 (52.0, 63.0)	60.0 (52.0, 60.0)	56.0 (52.0, 60.0)	56.0 (52.0, 60.0)	0.968
Anxiety problems	59.0 (54.0, 63.0)	59.0 (52.5, 65.0)	59.0 (51.0, 65.0)	63.0 (56.0, 70.0)	59.0 (51.0, 67.0)	59.0 (51.0, 65.0)	0.968
Somatic problems	56.0 (50.0, 61.0)	57.0 (50.0, 61.0)	50.0 (50.0, 61.0)	56.0 (50.0, 61.0)	56.0 (50.0, 61.0)	56.0 (50.0, 61.0)	0.827
ADHD problems	53.0 (50.2, 58.0)	51.0 (50.0, 55.5)	52.0 (50.0, 58.0)	55.0 (51.2, 62.2)	51.0 (50.0, 56.0)	52.0 (50.0, 57.5)	0.604
Oppositional defiant problems	55.0 (51.0, 59.0)	51.0 (51.0, 53.5)	52.0 (51.0, 55.0)	55.0 (51.0, 58.8)	52.0 (51.0, 55.0)	52.0 (51.0, 55.0)	0.968
Conduct problems	52.0 (50.0, 56.0)	52.0 (50.0, 56.0)	52.0 (50.0, 56.0)	52.0 (50.0, 56.8)	51.0 (50.0, 54.0)	52.0 (50.0, 56.0)	0.968
**SRS**							
SRS total T score	56.0 (51.0, 61.0)	54.0 (51.0, 59.0)	51.0 (44.5, 56.5)	54.0 (47.0, 61.5)	52.0 (47.0, 60.0)	54.0 (48.0, 60.0)	0.218

Total and divided by area CBCL scales and SRS total score mean (SD) parents report with Kruskal–Wallis rank-sum test p-values.

Bold values correspond to p-value < 0.05.

**Table 4:** Total and divided by area teacher CBCL scales and SRS total score.

	Tamburi (N = 78)	PaoloVI (N = 27)	Taranto (N = 59)	Statte (N = 30)	Talsano (N = 105)	Total (N = 299)	p value
**Empirically based scales**							
Anxious/depressed	**52.0 (50.0, 57.0)**	**56.0 (52.0, 60.5)**	**53.0 (51.0, 57.0)**	**50.5 (50.0, 51.8)**	**51.0 (50.0, 56.0)**	**52.0 (50.0, 57.0)**	**0.002**
Withdrawn/depressed	**53.0 (50.0, 57.0)**	**57.0 (53.0, 64.0)**	**53.0 (50.0, 60.0)**	**50.0 (50.0, 53.0)**	**50.0 (50.0, 57.0)**	**53.0 (50.0, 57.0)**	**0.013**
Somatic complaints	**50.0 (50.0, 57.0)**	**57.5 (50.0, 62.0)**	**50.0 (50.0, 50.0)**	**50.0 (50.0, 50.0)**	**50.0 (50.0, 50.0)**	**50.0 (50.0, 57.0)**	**0.001**
Social problems	50.0 (50.0, 54.0)	50.0 (50.0, 55.5)	50.0 (50.0, 54.0)	50.0 (50.0, 50.0)	50.0 (50.0, 53.0)	50.0 (50.0, 54.0)	0.207
Thought problems	50.0 (50.0, 50.0)	50.0 (50.0, 50.0)	50.0 (50.0, 50.0)	50.0 (50.0, 50.0)	50.0 (50.0, 50.0)	50.0 (50.0, 50.0)	0.260
Attention problems	50.0 (50.0, 53.0)	50.0 (50.0, 57.8)	50.0 (50.0, 51.0)	50.0 (50.0, 51.8)	50.0 (50.0, 51.0)	50.0 (50.0, 52.0)	0.260
Rule-breaking behavior	**50.0 (50.0, 55.0)**	**51.5 (50.0, 61.2)**	**50.0 (50.0, 50.0)**	**50.0 (50.0, 50.0)**	**50.0 (50.0, 51.5)**	**50.0 (50.0, 53.0)**	**0.037**
Aggressive behavior	**50.0 (50.0, 56.0)**	**52.0 (50.0, 59.5)**	**50.0 (50.0, 53.0)**	**50.0 (50.0, 51.0)**	**50.0 (50.0, 53.0)**	**50.0 (50.0, 53.0)**	**0.183**
Internalizing broad band score	**50.0 (44.0, 58.0)**	**56.0 (53.0, 63.8)**	**52.0 (47.0, 58.0)**	**46.0 (38.0, 51.0)**	**48.0 (38.0, 55.5)**	**51.0 (44.0, 58.0)**	**0.001**
Externalizing broad band score	48.0 (43.0, 55.0)	51.5 (43.0, 60.0)	48.0 (43.0, 53.5)	43.0 (43.0, 48.0)	43.0 (42.0, 52.0)	43.0 (43.0, 54.0)	0.067
Total problems score	**49.0 (43.2, 55.0)**	**49.5 (45.2, 62.8)**	**49.0 (43.5, 53.0)**	**43.0 (38.2, 50.0)**	**47.0 (41.0, 51.5)**	**47.0 (42.0, 53.0)**	**0.028**
**DSM-oriented scales**							0.260
Affective problems	50.0 (50.0, 55.0)	50.0 (50.0, 63.0)	50.0 (50.0, 54.5)	50.0 (50.0, 50.0)	50.0 (50.0, 50.0)	50.0 (50.0, 54.0)	0.024
Anxiety problems	**56.0 (50.0, 61.0)**	**58.5 (56.0, 65.0)**	**56.0 (50.0, 61.0)**	**50.0 (50.0, 56.0)**	**56.0 (50.0, 61.0)**	**56.0 (50.0, 61.0)**	**0.013**
Somatic problems	50.0 (50.0, 57.0)	50.0 (50.0, 63.0)	50.0 (50.0, 50.0)	50.0 (50.0, 50.0)	50.0 (50.0, 50.0)	50.0 (50.0, 50.0)	0.275
ADHD problems	50.0 (50.0, 54.0)	50.0 (50.0, 57.5)	50.0 (50.0, 52.0)	50.0 (50.0, 52.8)	50.0 (50.0, 52.0)	50.0 (50.0, 52.2)	0.260
Oppositional defiant problems	50.0 (50.0, 54.0)	50.0 (50.0, 56.0)	50.0 (50.0, 54.0)	50.0 (50.0, 50.0)	50.0 (50.0, 50.0)	50.0 (50.0, 54.0)	0.226
Conduct problems	**50.0 (50.0, 50.0)**	**50.0 (50.0, 58.5)**	**50.0 (50.0, 50.0)**	**50.0 (50.0, 50.0)**	**50.0 (50.0, 50.0)**	**50.0 (50.0, 50.0)**	**0.002**
**SRS**							
SRS total T score	**52.0 (46.2, 56.8)**	**53.0 (48.0, 62.0)**	**49.0 (45.0, 53.5)**	**48.5 (45.2, 55.8)**	**47.0 (44.0, 51.5)**	**50.0 (45.0, 55.0)**	**0.014**

Total and divided by area CBCL scales and SRS total score mean (SD) teachers report with Kruskal–Wallis rank-sum test p-values.

Bold values correspond to p-value < 0.05.

We found significant differences among the areas of residence for social problems, externalizing problems, oppositional defiant problems and the SRS total score reported by parents while for the teachers report there was a significant difference in all the scores apart from the thought problems, attention problems, affective problems, ADHD problems and oppositional defiant problems. Overall, we are able to see higher score values for the areas at closer distance from the emission source compared to those at greater distance.

Multicollinearity analysis was performed when multivariable models were applied: low VIF were observed for all the models fitted for each outcome (all VIF < 5). The marginal effects of the neurotoxic elements and 95% CI of the censored regression for the CBCL sub-scales are shown in Table 5 (only significant results were displayed). Significant positive association were found between blood Pb (BPb) and social problems and aggressive behavior scales while UAs has a greater impact on the anxious depressed, somatic complaints, attention problems and rule breaking behavior scales. The estimated effects of each models were at the net of sex, age, SES, distance from the source, IQ, exposure to passive smoking and HOME total score. In Table 5 are also reported the results of linear mixed effect models. In this case only BPb has a statistically significant impact on the externalizing and total problems scales of the CBCL, while UAs has a marginally significant association with total problems and the SRS total score. In this case the models were adjusted for the same covariates as the previous ones, but the random effects allowed to consider the nested structure of the data (subjects, schools and areas of residence). Overall, we were able to test that higher BPb or UAs concentrations are associated with increased neurobehavioral problems. All the other elements did not appear to have a statistically significant association with children neurobehavior. Semipartial correlations applied to multivariable models confirmed the results obtained and displayed in Table 5 and 6 showing higher R^2^ for BPb when considering the social problems, aggressive behavior, externalizing and total problems as dependent variables while UAs significantly increased the explained amount of variability of anxious depressed, somatic complaints, attention problems and rule breaking behavior (the proportion of explained variance associated to BPb and UAs was greater than 7% in all these cases).

**Table 5:** Tobit regression and linear mixed effects model to test neurotoxic chemical association with CBCL SRS scores.

	BPb	HMn	HHg	UAs	UCd	BSe
**Tobit regression**						
Anxious depressed	0.8 (− 0.8, 2.4)	− 0.5 (− 1.7, 0.6)	− 0.7 (− 1.7, 0.3)	**0.8 (0.1, 1.5)**	− 0.9 (− 1.7, 0.02)	− 0.6 (− 4.7, 3.5)
Somatic complaints	0.3 (− 2.2, 2.8)	0.6 (− 0.9, 2.0)	− 1.3 (− 2.7, 0.1)	**1.5 (0.1, 2.9)**	− 1.6 (− 3.2, − 0.01)	− 0.9 (− 6.5, 4.8)
Social problems	**1.5 (0.01, 2.9)**	− 0.1 (− 1.0, 0.9)	− 0.1 (− 1.0, 0.7)	0.04 (− 0.8, 0.9)	− 0.1 (− 1.0, 0.7)	0.3 (− 3.3, 3.9)
Attention problems	1.1 (− 0.5, 2.6)	− 0.2 (− 1.1, 0.8)	0.2 (− 0.7, 1.1)	**0.9 (0.2, 1.7)**	− 0.9 (− 1.8, 0.1)	− 0.6 (− 4.9, 3.6)
Rule breaking behavior	1.3 (− 0.3, 2.9)	− 0.3 (− 1.3, 0.7)	− 0.5 (− 1.3, 0.4)	**0.9 (0.2, 1.7)**	− 0.3 (− 1.2, 0.7)	− 0.2 (− 4.5, 4.1)
Aggressive Behavior	**2.2 (0.5, 4.0)**	0.1 (− 1.1, 1.1)	0.3 (− 0.8, 1.3)	0.3 (− 0.6, 1.1)	− 0.5 (− 1.5, 0.5)	− 1.2 (− 5.5, 3.2)
**Linear mixed effects model**						
Externalizing problems	**1.8 (0.1, 3.5)**	0.03 (− 1.0, 1.0)	0.04 (− 0.9, 1.0)	0.4 (− 0.4, 1.2)	− 0.5 (− 1.5, 0.5)	− 0.7 (− 4.8, 3.4)
Total problems	**1.8 (0.1, 3.6)**	− 0.001 (− 1.0, 1.0)	− 0.1 (− 1.0, 0.9)	0.7^a^ (− 0.1, 1.5)	− 1.0^a^ (− 2.1, 0.01)	− 1.1 (− 5.3, 3.2)
SRS total T score	1.3 (− 0.6, 3.2)	0.004 (− 1.1, 1.1)	− 0.6 (− 1.7, 0.4)	0.8^a^ (− 0.1, 1.7)	− 0.6 (− 1.7, 0.5)	− 1.4 (− 6.1, 3.3)

Tobit marginal effect coefficients (95% CI estimated with robust standard errors) and linear mixed effects model results (with participants (using both teachers and parents report), school and area of residence considered as random effects with a nested structure) for the association between neurotoxic chemicals and CBCL and SRS scores. Models were adjusted for sex, age, SES, distance from the exposure source, child IQ, passive smoking and HOME total score.

Bold values correspond to p-value < 0.05.

^a^Correspond to p-value < 0.1.

**Table 6:** Association between BPb and UAs interaction and externalizing and total problems and SRS total score.

As/Pb percentiles	Externalizing problems		Total problems		SRS total T score	
	Pb trend	As trend	Pb trend	As trend	Pb trend	As trend
10th	0.1 (− 0.9, 1.1)	− 0.4 (− 1.5, 0.8)	− 0.1 (− 1.1, 0.9)	− 0.3 (− 1.5, 0.9)	0.2 (− 0.9, 1.3)	0.4 (− 0.9, 1.7)
25th	0.4 (− 0.4, 1.2)	− 0.04 (− 1.0, 0.9)	0.3 (− 0.6, 1.1)	0.1 (− 0.9, 1.1)	0.3 (-0.6, 1.3)	0.6 (− 0.5, 1.6)
40th	0.6 (− 0.2, 1.3)	0.2 (− 0.6, 1.0)	0.6 (− 0.2, 1.3)	0.5 (− 0.4, 1.3)	0.4 (− 0.4, 1.3)	0.7 (− 0.2, 1.6)
50th	**0.7 (0.01, 1.4)**	0.4 (− 0.4, 1.2)	**0.8 (0.02, 1.5)**	0.7 (− 0.1, 1.5)	0.5 (− 0.3, 1.3)	0.8 (− 0.1, 1.7)
60th	**0.9 (0.1, 1.6)**	0.6 (− 0.2, 1.4)	**1.0 (0.2, 1.7)**	**0.9 (0.1, 1.8)**	0.6 (− 0.2, 1.4)	0.9 (− 0.01, 1.8)
75th	**1.1 (0.3, 1.9)**	0.8 (− 0.1, 1.7)	**1.3 (0.5, 2.2)**	**1.3 (0.4, 2.2)**	0.8 (− 0.2, 1.7)	**1.1 (0.04, 2.1)**
90th	**1.8 (0.5, 3.1)**	**1.3 (0.1, 2.4)**	**2.2 (0.9, 3.5)**	**1.9 (0.7, 3.1)**	1.1 (− 0.3, 2.6)	1.3 (− 0.03, 2.7)

Association between BPb and UAs and CBCL externalizing and total problems and SRS total T score when adding the interaction term between Pb and As. All models included all metal concentrations and were adjusted for sex, age, SES, distance from the exposure source, child IQ, passive smoking and HOME total score.

Bold character correspond to p-value < 0.05.

**Figure 1:**
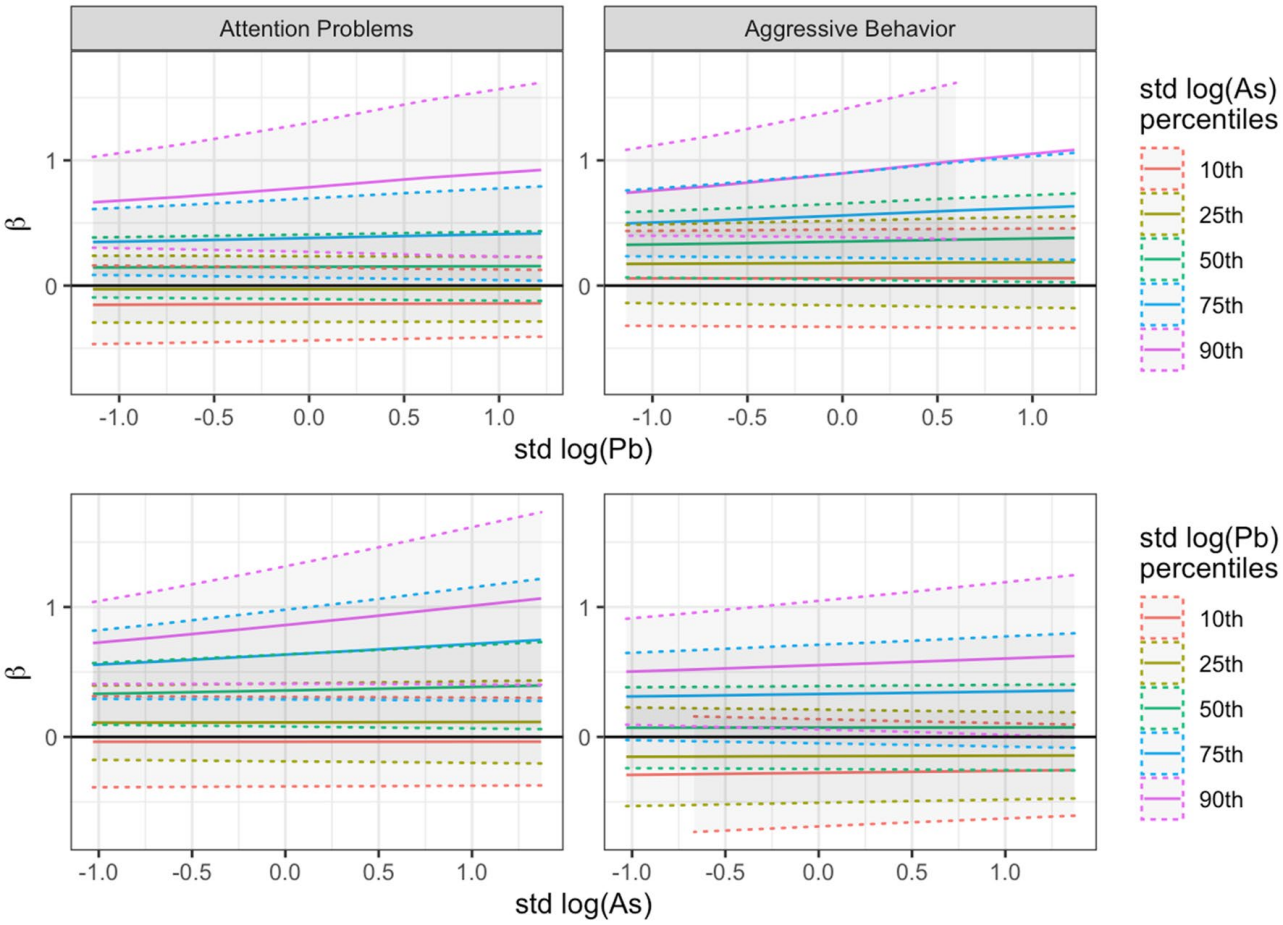
BPb and UAs interaction association with aggressive behavior and attention problems. Association between BPb and UAs interaction and aggressive behavior (first column) and attention problems (second column). In each box the change of the β parameter is represented as a function of BPb (first row) or UAs (second row) concentration. Each line shows how the β coefficient varies across the different levels of the interacting metal (UAs for the first row and BPb for the second row).

We finally introduced interaction terms between pairs of metals but only BPb and UAs showed a synergistic effect on child behavior. Figure 1 shows the effects of the interaction between BPb and UAs on aggressive behavior and attention problems. The first column of Fig. 1 shows the associations of both BPb (first row) and UAs (second row) with attention problems: for both metals we can see higher values of the β parameter at higher metal concentrations, moreover the effect becomes significant when at higher concentration levels of the interacting element. Specifically, BPb has a significant positive association with attention problems when UAs reaches its higher concentration levels (the black solid line corresponding to β = 0 is outside the CI when As is at the 75th and 90th percentiles). The same is for UAs where we can see a similar trend that reaches a significant association with attention problems even at lower BPb levels: the β parameter is significantly different from 0 starting from a concentration level of BPb corresponding to the median value. We can observe similar trends for aggressive behavior with a significant association with Pb, while As reaches statistical significance only for simultaneous very high Pb exposure (corresponding to the BPb 90th percentile). The results for anxiety and depression, somatic complaints, social problems and rule breaking behavior, showing association respectively with Pb and As, are reported in Figs. S1 and S2 of Supplementary Material.

When considering the externalizing problems, the total problems and the SRS total T score and adding the interaction term between BPb and UAs (Table 6), a significant conditional effect of both Pb and As is also observed for increasing UAs and BPb concentration respectively. BPb reached significance in the association with externalizing and total problems when UAs was at least equal to its median value. UAs showed a significant association with externalizing problems when BPb was as high as the 90th percentile, with total problems when BPb was equal or greater than the 60th percentile and with SRS total T score when BPb reached the 75th percentile.

**Figure 2:**
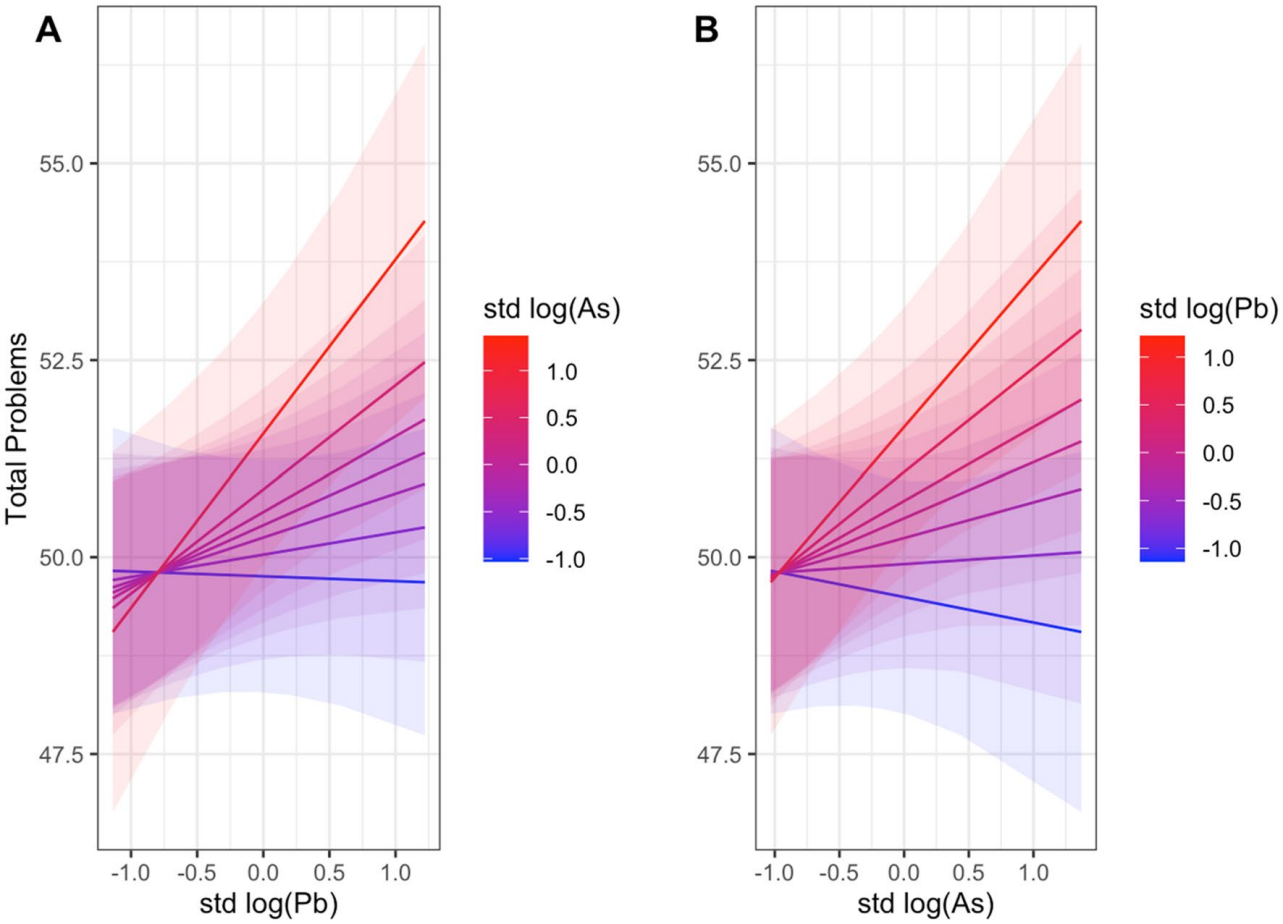
BPb and UAs interaction association with total problems. BPb and UAs association with CBCL total problems when an interaction term between the two metal concentrations is included in the linear mixed effects model. Blue color corresponds to low level of Pb or As concentrations while red corresponds to high concentration levels.

We represented the effect of the interaction between BPb and UAs on CBCL total problems score in Fig. 2, to provide a better representation of how the association between the outcome and the BPb and UAs changed at increasing levels of the respective interacting element. A clear pattern of an increased metal impact on behavioral total problems at higher levels of both the element of interest and the interacting metal can be appreciated. The results for externalizing problems and SRS total T score are shown in Figs. S1 and S2 respectively of Supplementary Material.

## Discussion

The aim of this study was to explore the association between the exposure to elements potentially neurotoxic and the neurobehavioral outcomes. We were able to identify Pb and As and their interaction as important risk factors in increasing the likelihood of neurobehavioral problems among the children residing in the province of Taranto.

Increased hyperactivity and psychopathological traits, impairment of social behavior and higher risk of autism were detected in the neighborhoods of Tamburi and Paolo VI, which are located at closer distance from the industrial emission. We were able to find major differences among the areas of residence considering the teacher reports compared to the parent ones. Along with a decrease of the neurobehavioral scores at higher distance from the plant (meaning fewer behavioral problems in the areas at greater distance) we observed negative trends also for understudied element concentrations but not for BSe and HHg. However, the first element is known to have a protective effect on neurodevelopment while the second can be confounded by fish intake; the lack of this information is a limitation of the current study.

When looking at the association between the biomarkers and the neurobehavioral outcomes in a multivariable model we were able to find significant association with BPb and UAs even adjusting for SES and the distance from the plant. BPb mainly influenced social problems, aggressive behavior, externalizing and total problems. UAs showed an impact on anxiety, depression, somatic problems, attention problems and rule breaking behavior beside a significant association with autism (measured through the SRS T total score). This is in line with the U.S. Environmental Protection Agency’s (EPA) priority list of hazardous substances where As and Pb are ranked as first and second respectively.

Because of the absence of multicollinearity among metals, the skewed distribution as well as the multilevel structure of the dependent variables we opted for the application of linear mixed effect models and censored regressions instead of more recent environmental mixture models like Weighted Quantile Sum (WQS) regression^32^ or Bayesian Kernel Machine Regression (BKMR)^33^. This choice did not allow us to estimate an overall effect of metal mixture as in WQS or BKMR, however we were still able to find the association between each chemical and the outcomes at the net of the exposure to the other elements and to account for interactions among metals. Through the inclusion of an interaction term between pairs of metals we were able to observe a multiplicative effect of Pb and As on child behavior. In particular a synergistic effect of the two chemicals was highlighted in the association with attention problems, aggressive behavior, externalizing and total problems. The mechanism of neurotoxicity of these two elements are different, as they involve the inhibition of some cellular elements and the rising of apoptotic factors causing the neuronal cell death. However, the mechanism through which the mixture of metals causes neuronal damage is still unclear and needs further exploration^31^.

Through this and the previous work Lucchini et al. 2019, we were able to observe an association of Pb on both behavior and cognition. These adverse effects of Pb were also showed by other studies^34,35^ and were associated to alterations in DNA and chromosomal integrity, interaction with proteins^36^ and alteration to cellular redox status^37^.

The biomonitoring measurement did not show elevated levels compared to the available standards or to other similar community studies^38–52^. Nevertheless, we were able to find a significant association between the exposure and the outcomes. This result highlights the importance of considering the possible interactions among pollutants that can amplify the effects of the exposure even at low levels as already pointed out by previous studies that showed how co-occurring metal exposure may contribute to a greater risk of autism^29^ and behavioral outcomes^27,28,30^. However, our data mainly reflect current exposure, and cannot exclude previous higher exposure levels that may have occurred in the past years when the plant was at higher production levels than today.

This is a complementary analysis to the previous study^38^ where we found an association between the exposures to neurotoxic elements and neurocognitive abilities among children residing in the Taranto area. In this study we considered the same population but focusing on neurobehavioral aspects. This study also highlights how disadvantaged areas are at increased risk of neurobehavioral problems besides the risk of lower neurocognitive abilities as already pointed out in Lucchini et al. 2019.

We observed a poor agreement between parent and teacher evaluation, however even if there was no available tendency to lie measure, thanks to the availability of both the parent and teacher reports we had more robust assessment of children neurobehavioral problems because of the different perception and expectation of the two persons being interviewed. Since teacher reports can only amplify the effect of chemicals on behavior within the same sex^24^, the double report from both teachers and parents can avoid to incur in this situation.

The study has also some limitation: we were not able to account for potential selection bias which may have been generated for the low participation rate; due to the cross-sectional nature of the study we cannot attribute a causative role to the neurotoxic elements on child behavior; the biomonitoring covered only current exposure excluding prenatal and postnatal periods which are considered vulnerable exposure windows, in particular the lack of information about previous exposure during the higher production levels of the plant is a limit in identifying the impact that this chemicals can have on the considered outcomes; UAs is a marker of short term exposure, the unavailability of As in hair or nails which are biomarkers of longer-term burden, is a limitation; we could also not consider the effect that concurrent exposure to other factors such as organic compounds can have on neurodevelopment; although we adjusted for several confounders there still was missing information like parental relations; finally the sample size was probably not sufficient to entirely detect the association of metals and their interactions on neurobehavior.

In this work we found that As and Pb can affect school-children behavior by increasing the risk of neurobehavioral problems and autism and have a synergistic effect on some child behavioral problems. Through this study we highlighted the importance of considering the interactions among pollutants since it can amplify the effect of the exposure even at low levels. Further work is needed to address the neurological effect of the exposure to mixtures of metals considering their interactions.

## Methods

### Study area and population

The study area, the sample selection and the recruitment strategies were already described in previous works^38,53^. Briefly, the study area is the city of Taranto, South Italy, where a wide industrial pole including one of the largest steel producers in Europe operates since many decades, causing emission of toxic elements and many other chemical compounds in a wide surrounding area. 12 primary schools located in the 5 sub-areas by incremental distance from the industrial pole were selected by the enrollment procedures.

A total of 700 children-parent pairs were invited to participate on voluntary bases to the study. Children from 6 to 11 years-old were enrolled through the public school system according to a community-based participatory approach that involved the community of Taranto. The aims and methodology of the study were explained through ad hoc meetings and the availability of brochures for teachers, parents and children. 432 subjects agreed to participate to the study showing a 62% participation rate. Informed consent was obtained from all parents. Subjects who agreed to participate filled in a screening questionnaire for the evaluation of inclusion criteria which were being born and raised in the target study areas, and having carried the entire pregnancy period of the mother in the same area at the time of recruitment, and the exclusion criteria: familiarity of neurodegenerative diseases, diagnosis/treatments for neurological and psychiatric illnesses, hepatic or biliary diseases, dysmetabolic diseases, endocrine disorders, kidney disorders, previous total parenteral nutrition and uncorrected visual defects. Overall 299 children matched the inclusion criteria reaching the minimum sample size estimated by power calculation where the effect of the 6 metals was considered on cognitive outcomes hypothesizing around 1.4% explanation of the outcome variability for each element based on a previous study^50^ and allowing for interactions and multiple testing but not considering the strata; in particular, for each school an average of 27 students were recruited ranging from a minimum of 16 to a maximum of 56. The study design, the information about the study aims and the forms for informed consent had been reviewed and approved by the ethical committees of the local Public Health Agency of Brindisi. All methods were performed in accordance with the relevant guidelines and regulations.

### Elements biomonitoring

Whole blood, urine and hair were collected in the schools on a day before the neuropsychological testing. High Resolution-Inductively Coupled Plasma Mass Spectrometry (HR-ICP-MS) (ElementII, Thermo Scientific, Bremen, Germany) was used to measures in whole BPb and BSe, in UAs and urinary Cd (UCd), and in hair Mn (HMn)^54,55^. In addition, HHg was quantified by thermal decomposition amalgamation atomic absorption spectrometry (TDA-AAS) using a Direct Mercury Analyzer (DMA-80 TRICELL, FKV, Bergamo, Italy)^56^. Blood and urine were refrigerated at − 20 °C. Both analytical procedures have been extensively described in previous papers, including the quality control aspects, in Ruggieri et al. 2016 and Alimonti et al. 2015 for HR-ICP-MS and Domanico et al. 2017 for DMA analysis.

### Psychological assessment, adult survey about the child

The neuropsychological assessment was conducted by four neuropsychologists who performed periodical intercalibration of testing procedure and scoring. The Child Behavior Checklist (CBCL), administered to the main teacher and to the mother^57^ was considered to identify problem behavior in children. This test measures eight empirically-based syndrome scales: Aggressive Behavior, Anxious/Depressed, Attention Problems, Rule-Breaking Behavior, Somatic Complaints, Social Problems, Thought Problems, Withdrawn/Depressed. All these scales can be combined in the Internalizing problems and the Externalizing problem scores which can be summarized in turn in the Total problem score. The Diagnostic and Statistical Manual of Mental Disorders (DSM) oriented scales were also considered: Affective Problems, Anxiety Problems, Somatic Problems, ADHD Problems, Oppositional Defiant Problems, Conduct Problems. The Social Responsiveness Scale (SRS)^58^ was administered to measure autistic traits as observed by the main teacher and by the mother through a 65 questions survey. For both CBCL and SRS scores a lower value means fewer behavioral problems.

### Psychological assessment, child performance

The Wechsler Intelligence Scale for Children edition IV (WISC-IV)^59^ (the Italian version of the most recent WISC-V was not yet available) for children’s cognitive assessment was also taken by the participants.

### Sociodemographic and lifestyle data

Sociodemographic data including age, sex, SES, area of residence and distance from the source were collected from participants through the administration of questionnaires to parents. Parental age, level of education, occupational level and work-related stress perception were considered to categorize each subject in three education and occupational levels (low, medium, high) through whose combination a SES variable with as many levels was built^60^. Distance from the source was measured as the distance from the closest point of the polygon delimiting the industrial site responsible of the major amount of the emissions. Lifestyle information was also collected like exposure to passive smoking at home and cognitive stimulation via the Home Observation for Measurement of the Environment (HOME) questionnaire^61^.

### Statistical analysis

Descriptive statistics were reported for all the variables of interest for the overall sample size and stratified by area of residence (Tamburi, Statte, Paolo VI, Taranto, Talsano). Frequencies and proportions were used for categorical variables, median and interquartile range for continuous variables. Preliminary comparison between the study sub-sites was performed with non-parametric Kruskal–Wallis rank-sum test. The correlation among metal concentrations was measured through the Spearman correlation coefficient.

Biomarker statistics were reported as in Lucchini et al.^38^: median, 1st and 3rd quartiles were shown alongside the linear regression coefficient and 95% confidence intervals (CI) related to the association between the distance to the emission source and the biomarkers after having log-transformed the metal concentrations to obtain a normal distribution and after having applied cubic splines to the distance from the source to asses a linear trend.

The homogeneity between parent and teacher scores was assessed through the intraclass correlation coefficient (ICC). After checking for skewness and kurtosis of each neurobehavioral outcome, the association between exposure to multiple elements and the combined syndrome scales (internalizing, externalizing and total problems) and SRS scores was tested through a linear mixed effects model allowing to consider both parent and teacher scores with participants, school and area of residence considered as random effects with a nested structure. A censored regression^62^ was applied to the lower level empirically-based syndrome and the DSM oriented scales because of the censored and skewed data (the score is included between 50 and 100 and there was a mass point at the lower limit). Robust estimates of the standard errors clustered by subject were considered allowing to include both scores obtained from the questionnaire filled out by parents and teachers. For the censored regression we reported the marginal effect (the effects on the actual bounded dependent variables) at the mean value of the covariates since significance did not change both for lower or higher values. Multicollinearity was assessed in multivariable models through the variance inflation factor (VIF) while semipartial correlation was estimated to assess which variable explained the higher proportion of variance for each outcome. An interaction term between pairs of metal was introduced (as the product of the two considered metals) in the linear mixed effect models and the censored regressions. The marginal effect of each element at increasing level of the interacting chemical was shown for both models. In particular for the linear mixed effects model we reported the total effect of each metal on the considered outcome at different level of concentrations of the interacting element. In the case of the censored regression, since the parameter not only changes at different levels of the variable with which it interacts (due to the interaction term) but also with the variation of the variable with which it is associated (when considering the marginal effects), we represented the β parameters with their 95% CI as a dependent variable of the metal with which it was associated and at different levels of the interacting element.

We included in the analyses the values < LOD (Limit of Detection) (these were 1.6% of the results, only for UCd) to not loose statistical power.

All models were adjusted for sex, age, SES, distance from the exposure source, child IQ, exposure to passive smoking and the total HOME score as potential confounders. All statistical tests were two sided and assumed a 5% significance level after applying Hochberg adjusted p-values to account for multiple testing for Kruskal Wallis test, while Tukey adjustment was considered for marginal effects. All analyses were performed with R 4.0.2^63^ and Stata 15^64^.

### Ethics approval and consent to participate

This study obtained the approval of the Ethical Committee of Local Public Health (ASL, Azienda Sanitaria Locale) of Brindisi, with the jurisdiction on Taranto province.

## Supplementary Information

Supplementary Information.

## Data Availability

The datasets generated during and analysed during the current study are available from the corresponding author on reasonable request.

## Abbreviations

ADHD: Attention Deficit Hyperactivity Disorder
CDC: Centers for Disease Control and Prevention
Pb: Lead
Hg: Mercury
Cd: Cadmium
Mn: Manganese
As: Arsenic
Se: Selenium
HR-ICP-MS: High resolution-inductively coupled plasma mass spectrometry
BPb: Blood lead
BSe: Blood selenium
UAs: Urine arsenic
UCd: Urine cadmium
HMn: Hair manganese
HHg: Hair mercury
TDA-AAS: Thermal decomposition amalgamation atomic absorption spectrometry
CBCL: Child Behavior Checklist
DSM: Statistical Manual of Mental Disorders
SRS: Social Responsiveness Scale
WISC-IV: Wechsler Intelligence Scale for Children edition IV
SES: Socio-economic status
CI: Confidence intervals
LOD: Limit of detection
EPA: Environmental Protection Agency’s
