# Supplementary material for: The effects of the exposure to neurotoxic elements on Italian schoolchildren behavior
Source: Sci Rep. 2021 May 10;11:9898. doi: 10.1038/s41598-021-88969-z (PMC8110539; doi:10.1038/s41598-021-88969-z)

**THE EFFECTS OF THE EXPOSURE TO NEUROTOXIC ELEMENTS ON ITALIAN SCHOOLCHILDREN BEHAVIOR**

Stefano Renzetti^a,b,*^, Giuseppa Cagna^a^, Stefano Calza^b^, Michele Conversano^c^, Chiara Fedrighi^a^, Giovanni Forte^d^, Augusto Giorgino^c^, Stefano Guazzetti^a,f^, , Costanza Majorani^e^, Manuela Oppini^a^, Marco Peli^a,g^, Francesco Petrucci^d^, Anna Pino^d^, Donatella Placidi^a^, Oreste Senofonte^d^, Silvia Zoni^a^, Alessandro Alimonti^d^ Roberto G Lucchini^a,h^

1. Occupational Health, University of Brescia, Italy
2. Department of Molecular and Translational Medicine, University of Brescia, Italy
3. Department of Public Health, ASL, Taranto, Italy
4. Italian National Institute of Health, Department of Environment and Health, Rome, Italy
5. Italian National Institute of Health, National centre for Chemicals, Cosmetics and Consumer protection, Rome, Italy
6. Department of Public Health, Azienda USL - IRCCS, Reggio Emilia, Italy
7. Department of Civil, Environmental, Architectural Engineering and Mathematics, University of Brescia, Italy
8. Icahn School of Medicine at Mount Sinai, New York, USA

^*^Corresponding Author: Stefano Renzetti, Department of Occupational Health, University of Brescia, Piazzale Spedali Civili, 1, 25123 Brescia BS. stefano.renzetti@unibs.it Tel +390303717715

**Table1.** Spearman correlation matrix among all 6 metal concentrations.

|  | **UCd** | **UAs** | **BPb** | **BSe** | **HMn** |
| --- | --- | --- | --- | --- | --- |
| **UAs** | 0.253 |  |  |  |  |
| **BPb** | -0.047 | 0.0953 |  |  |  |
| **BSe** | -0.159 | 0.1081 | 0.058 |  |  |
| **HMn** | 0.065 | 0.109 | 0.056 | -0.046 |  |
| **HHg** | -0.133 | 0.276 | -0.035 | 0.173 | 0.049 |

**Figure 1. Effects of BPb on anxious depressed, somatic complaints, social problems and rule breaking behavior**. Effects of BPb on anxious depressed, somatic complaints, social problems and rule breaking behavior when considering the interaction with UAs. In each box the change of the β parameter is represented as a function of BPb concentration. Each line shows how the β varies across the different levels of UAs (the interacting metal).


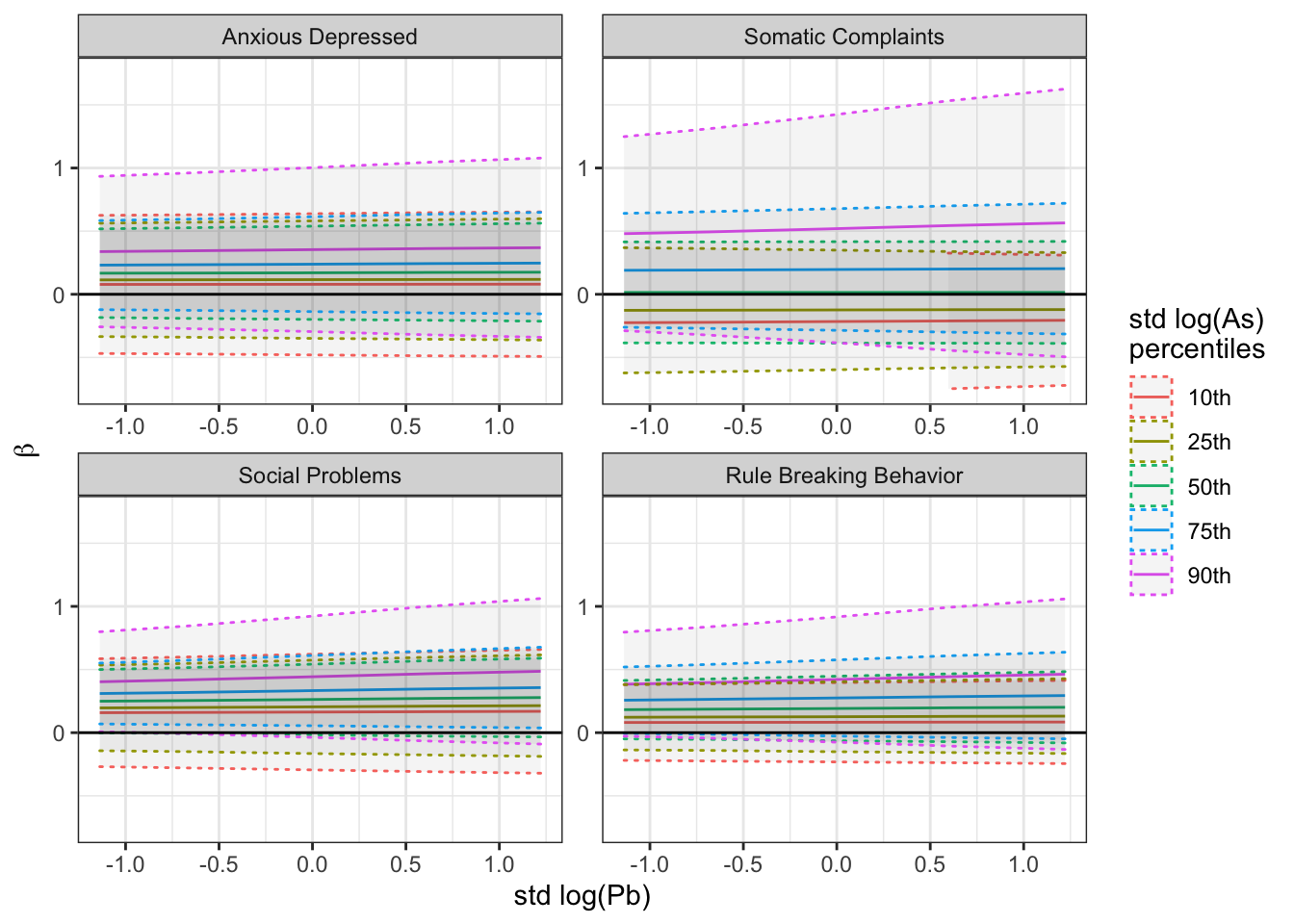


**Figure 2. Effects of UAs on anxious depressed, somatic complaints, social problems and rule breaking behavior**. Effects of UAs on anxious depressed, somatic complaints, social problems and rule breaking behavior when considering the interaction with BPb. In each box the change of the β parameter is represented as a function of UAs concentration. Each line shows how the β varies across the different levels of BPb (the interacting metal).


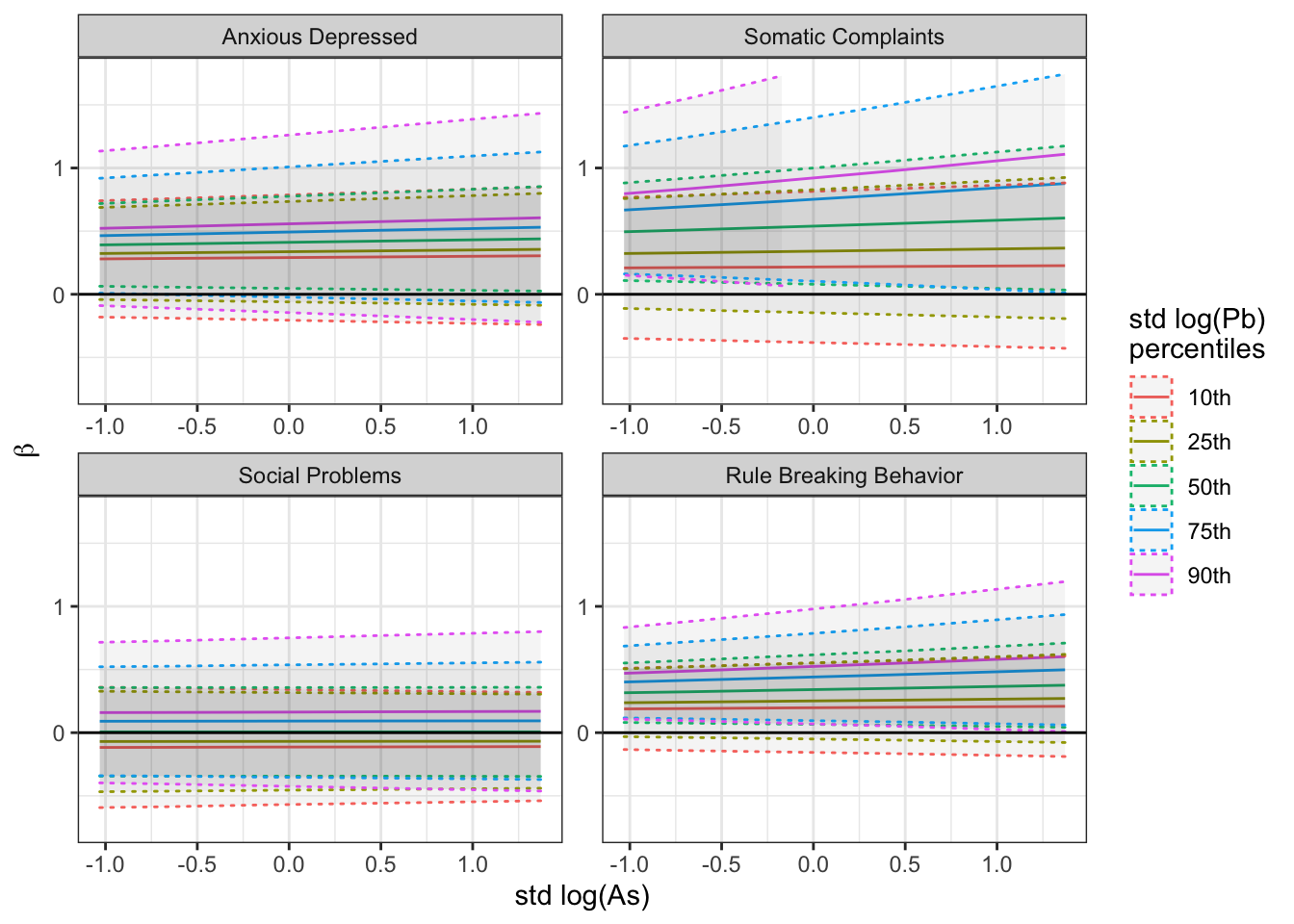


**Figure 3. BPb and UAs interaction effect on CBCL externalizing problems**. BPb and UAs effect on CBCL externalizing problems when an interaction term between the two metal concentrations is included in the linear mixed effects model. Blue color corresponds to low level of Pb or As concentrations while red corresponds to high concentration levels.


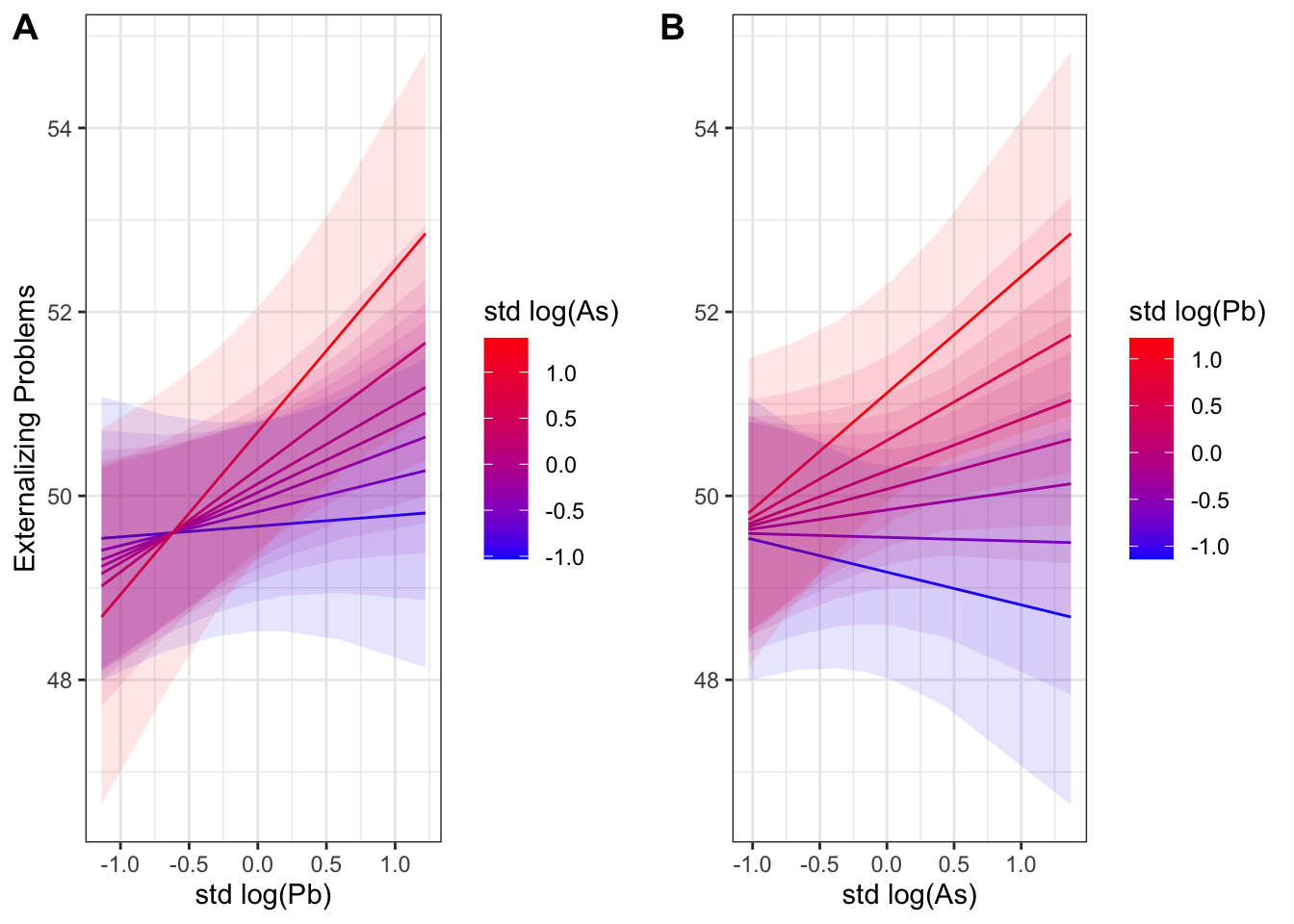


**Figure 4. BPb and UAs interaction effect on SRS total T score**. BPb and UAs effect on SRS total T score when an interaction term between the two metal concentrations is included in the linear mixed effects model. Blue color corresponds to low level of Pb or As concentrations while red corresponds to high concentration levels.


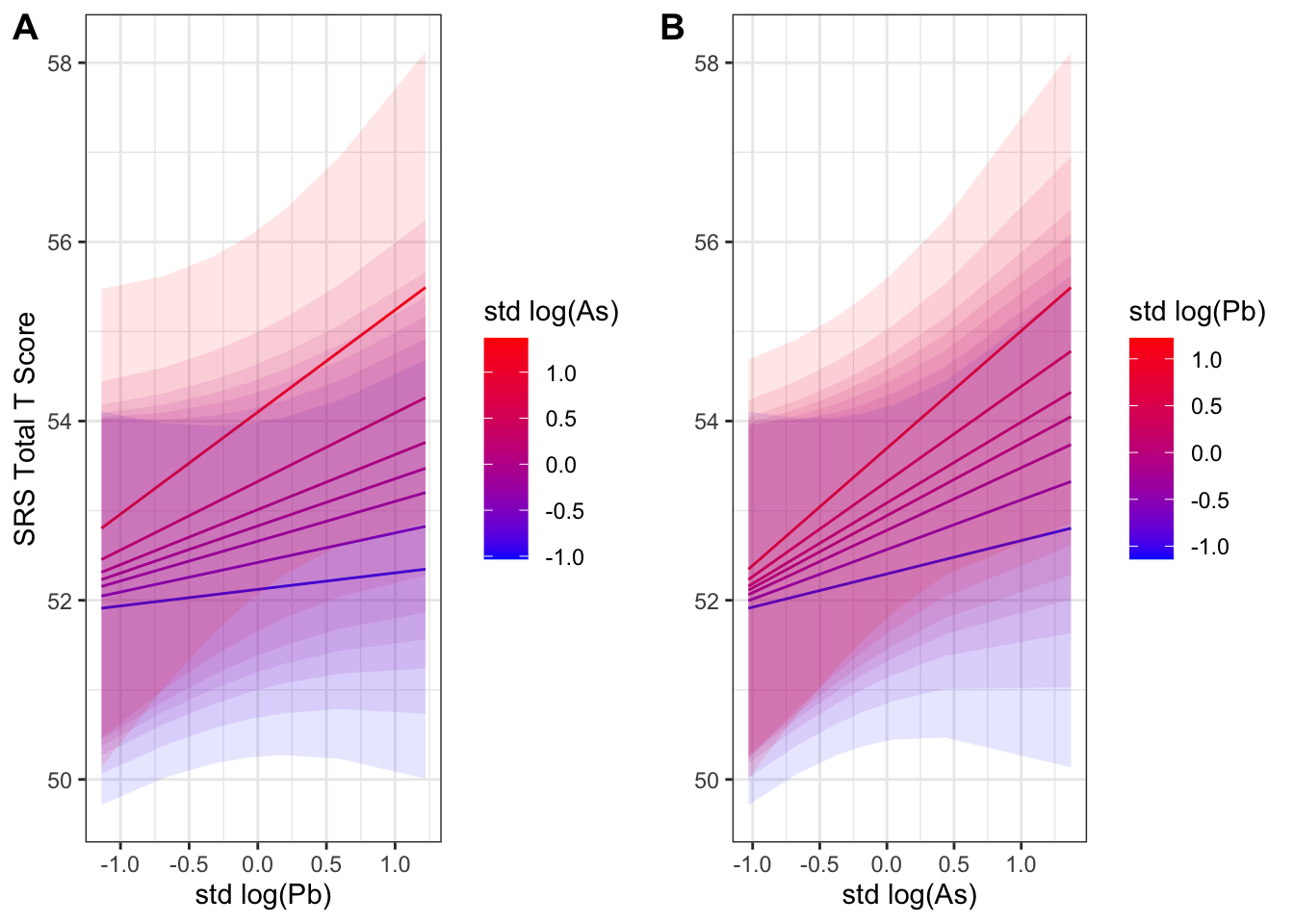

Supplement: Supplementary file 1 — Supplementary Information. [file 41598_2021_88969_MOESM1_ESM.docx]
